# Angiopoietin-2 is increased in sepsis and inversely associated with nitric oxide-dependent microvascular reactivity

**DOI:** 10.1186/cc9020

**Published:** 2010-05-18

**Authors:** Joshua S Davis, Tsin W Yeo, Kim A Piera, Tonia Woodberry, David S Celermajer, Dianne P Stephens, Nicholas M Anstey

**Affiliations:** 1 International Health Division, Menzies School of Health Research and Charles Darwin University, Ellengowan Drive, Casuarina, Darwin, NT 0810, Australia; 2 Department of Infectious Diseases, Royal Darwin Hospital, Rocklands Drive, Tiwi, Darwin, NT 0810, Australia; 3 Department of Medicine, University of Sydney and Department of Cardiology, Royal Prince Alfred Hospital, Missenden Road, Camperdown, Sydney, NSW 2006, Australia; 4 Intensive Care Unit, Royal Darwin Hospital, Rocklands Drive, Tiwi, Darwin, NT, 0810, Australia

## Abstract

**Introduction:**

Angiopoietin-2 (ang-2), an angiogenic peptide released by endothelial cell Weibel-Palade bodies (WPBs), increases endothelial activation and vascular permeability. Ang-2 is raised in severe sepsis but the mechanisms underlying this are not known. Nitric oxide (NO) inhibits WPB exocytosis, and bioavailability of endothelial NO is decreased in sepsis. We hypothesized that endothelial NO bioavailability would be inversely correlated with ang-2 concentrations in sepsis.

**Methods:**

Plasma ang-2, vascular endothelial growth factor (VEGF) and endothelial-active cytokines were assessed in 83 patients with early sepsis and 41 hospital controls, and related to reactive hyperaemia-peripheral arterial tonometry, RH-PAT, a measure of endothelial NO bioavailability.

**Results:**

Plasma Ang-2 was elevated in sepsis (median [interquartile range (IQR)], ng/ml: severe sepsis 12.4 [8.5-33.4], sepsis without organ failure 6.1 [5.0-10.4], controls 2.7 [2.2-3.6], *P *< 0.0001). It correlated inversely with RH-PAT (r = -0.38, *P *< 0.0001) and positively with IL-6 (r = 0.57, *P *< 0.0001) and degree of organ failure (sequential organ function assessment score) (r = 0.58, *P *< 0.0001). The correlation of ang-2 with RH-PAT persisted after controlling for sepsis severity. In a longitudinal mixed-effects model, recovery of RH-PAT over time was associated with decline in ang-2.

**Conclusions:**

Ang-2 is elevated in proportion to sepsis severity, and inversely correlated with NO-dependent microvascular reactivity. Impaired endothelial NO bioavailability may contribute to increased endothelial cell release of ang-2, endothelial activation and capillary leak. Agents that increase endothelial NO bioavailability or inhibit WPB exocytosis and/or Ang-2 activity may have therapeutic potential in sepsis.

## Introduction

Microvascular and endothelial dysfunction are central to the pathophysiology of sepsis, contributing to organ dysfunction even in the setting of normal post-resuscitation haemodynamics [1]. Angiopoietin-2 (ang-2), an angiogenic peptide, activates endothelial cells and increases vascular inflammation. It functions as an autocrine mediator of the endothelium and is stored predominantly in endothelial cells [2]. Ang-2 is a ligand of the tyrosine kinase receptor, Tie-2, and antagonises the angiopoietin 1-induced Tie-2 receptor autophosphorylation responsible for the maintenance of endothelial cell quiescence [3]. This results in endothelial cells being sensitized to the effects of pro-inflammatory cytokines and vascular endothelial growth factor (VEGF), resulting in a loss of endothelial cell quiescence and an increase in vascular activation and inflammation.

Levels of circulating ang-2 have been shown to be raised in human sepsis [4-6] and, more recently, to correlate with mortality [7-9] and pulmonary vascular leak [10,11]. Despite a growing interest in ang-2 in sepsis, the mechanisms underlying elevated ang-2 levels in patients with sepsis are unclear. Ang-2 is co-packaged with von Willebrand Factor (vWF) within endothelial cell Weibel-Palade bodies (WPBs) and is immediately released upon endothelial cell stimulation and WPB exocytosis [12]. *In-vitro *studies demonstrate that exocytosis of WPBs can be triggered by multiple secretagogues, including thrombin, histamine, epinephrine, VEGF, and hypoxia [13]. However, there are only two known inhibitors of WPB release: nitric oxide (NO) and hydrogen peroxide (H_2_O_2_), of which NO is thought to be the most important [14].

We have recently demonstrated impaired microvascular reactivity in patients with sepsis by reactive hyperaemia peripheral arterial tonometry (RH-PAT) [15], which is at least 50% NO-dependent and thus provides an estimate of endothelial NO bioavailability [16]. In contrast to earlier hypotheses suggesting major overproduction of NO in patients with sepsis [17], there is now increasing evidence that systemic NO production is normal or decreased in humans with sepsis [18,19]. Impaired endothelial NO bioavailability may underlie increased WPB exocytosis in sepsis, and thus the release of ang-2 from endothelial cells. However, the relation between endothelial NO bioavailability and measures of WPB release in sepsis has not been determined. We hypothesized that plasma ang-2 levels in patients with sepsis would be raised in proportion to disease severity and would be inversely related to endothelial NO bioavailability, as estimated by RH-PAT.

## Materials and methods

### Study design and setting

We performed a prospective observational study at a 350-bed teaching hospital in tropical Australia, with an 18-bed mixed ICU. Prior approval was obtained from the Human Research Ethics Committee of the Menzies School of Health Research and the Department of Health and Community Services. Written informed consent was obtained from all participants or their next of kin where necessary.

### Participants

The study subjects were adults (≥ 18 years) hospitalized with sepsis, who were enrolled in a previously reported study of microvascular reactivity; more detail of subject recruitment, patient characteristics and study procedures are provided in this paper [15]. Some of the data included in the current paper have been previously reported (RH-PAT index, intra-cellular adhesion molecule-1 [ICAM1], E-selectin and IL-6), but are included here for comparison with Ang-2. Sepsis was defined as a proven or suspected infection plus at least two criteria for the systemic inflammatory response syndrome (SIRS) present within the past four hours [20]. Septic patients were eligible for enrolment within 24 hours of their admission to the ICU, or within 36 hours of admission to the ward. Exclusion criteria were: coagulopathy (platelets ≤ 20 × 10^9^/L, activated partial thromboplastin time ≥ 70 seconds, international normalized ratio ≥ 2.0); smoking of tobacco within the preceding four hours; and receipt of intravenous nitrates. Control subjects were adults recruited from hospital patients with no clinical or laboratory evidence of inflammation or infection, and who had not met SIRS criteria within the past 30 days. Septic patients were prospectively classified as severe sepsis, or sepsis without organ failure. Severe sepsis was defined as sepsis with organ dysfunction or shock at the time of enrolment according to American College of Chest Physicians/Society of Critical Care Medicine consensus criteria [15,20].

### Laboratory assays

Venous blood was collected in lithium heparin tubes at baseline and two to four days later, and plasma was frozen at -80°C. Control patients had blood collected at baseline only. Plasma concentrations of VEGF, ICAM-1 and E-selectin were measured by ELISA (R&D Systems, Minneapolis, MN, USA), according to the manufacturer's instructions. The ELISA used to determine plasma Ang-2 concentrations (R&D Systems, Minneapolis, MN, USA) reports a lower limit of detection of 8.3 pg/ml (0.0083 ng/ml), with coefficients of variation for intra-assay and inter-assay precision of 4.2% and 7.4%, respectively.

IL-6 and TNFα were measured by flow cytometry using a cytokine bead array (CBA; BD Biosciences, Franklin Lakes, NJ, USA).

### Measurement of microvascular reactivity/endothelial NO bioavailability

Microvascular reactivity was measured at the bedside by RH-PAT (Itamar Medical, Caesarea, Israel), a non-invasive method of assessing endothelial function [21], which is at least 50% dependent on endothelial NO production [16]. We have previously reported internal validation and repeatability of RH-PAT in acute inflammatory states [22]. PAT was measured in a fingertip before and after a five-minute ischemic stress at the forearm, generating an RH-PAT index, normalized to the control arm, as previously described [15]. All studies were performed after resuscitation and at least an hour of hemodynamic stability in a quiet room at 25°C, with the patient in a recumbent position.

### Statistical methods

All analyses were hypothesis based and were specified *a priori*. Continuous variables were compared using Student's t-test/analysis of variance or Mann Whitney U test/Kruskal-Wallis test for parametric and non-parametric variables, respectively. Categorical variables were compared using Fisher's exact test. For the ELISA and CBA assays, values below the lower limit of detection were assigned a value of halfway between zero and the lower limit of detection for the purposes of analysis. Correlates with baseline Ang-2 were determined using Spearman's coefficient. For multivariate analysis, linear regression with backward selection was used. All independent variables with a Wald *P*-value of less than 0.10 on univariate analysis, or which were considered biologically important were included in the initial model. Variables with a Wald *P*-value of 0.05 or more were sequentially dropped from the model. The natural logarithm of Ang-2 was used as the dependent variable, because Ang-2 was right-skewed and log transformation lead to a normal distribution. To control for covariates in the relationship between Ang-2 and RH-PAT index, the covariate in question was added to a linear regression model with log Ang-2 as the independent variable and RH-PAT index as the dependent variable. To examine longitudinal correlations, linear mixed-effects models were used. A two-sided *P *value of less than 0.05 was considered significant. All analyses were performed using Stata version 10 (Stata Corp, College Station, TX, USA).

## Results

### Participants

Eighty five patients with sepsis and 45 control patients were enrolled in the study. Two sepsis patients and four controls were excluded from further analysis because they refused blood collection. Of the remaining 83 sepsis patients, 52 had organ dysfunction due to sepsis at baseline (severe sepsis group) and 31 did not (sepsis without organ failure). The three groups were well matched in terms of risk factors for endothelial dysfunction and other baseline characteristics (Table 1).

**Table 1:** Baseline characteristics of participants

	Severe sepsis	Sepsis without organ failure	Control	*P *value across all groups
**N**	52	31	41	
**Age (years)^a^**	52.8 (48.6-56.9)	50.8 (46.5-55.2)	47.3 (43.2-51.6)	NS^c^
**Male n (%)**	31 (60)	21(68)	28 (68)	NS^d^
**Diabetic n (%)**	17 (33)	7 (23)	13 (32)	NS^d^
**Smoker n (%)**	26 (50)	12 (39)	16 (39)	NS^d^
**IHD n (%)**	9 (17)	6 (19)	5 (12)	NS^d^
**On a statin n (%)**	13 (25)	9 (29)	11 (27)	NS^d^
**APACHE II^b^**	19 (15-25)	8 (5-11)		0.0001^e^
**SOFA score^b^**	6 (3-9)	1 (0-2)		0.0001^e^

a. Mean (95% confidence interval).

b. Median (Interquartile range).

c. By one-way analysis of variance.

d. By Fisher's exact test across all three groups.

e. By Kruskal-Wallis test.

APACHE II, Acute Physiology and Chronic Health Evaluation II score; IHD, ischemic heart disease; NS, not significant; SOFA, Sequential Organ Failure Assessment score.

### Baseline Ang-2 and VEGF

**Figure 1:**
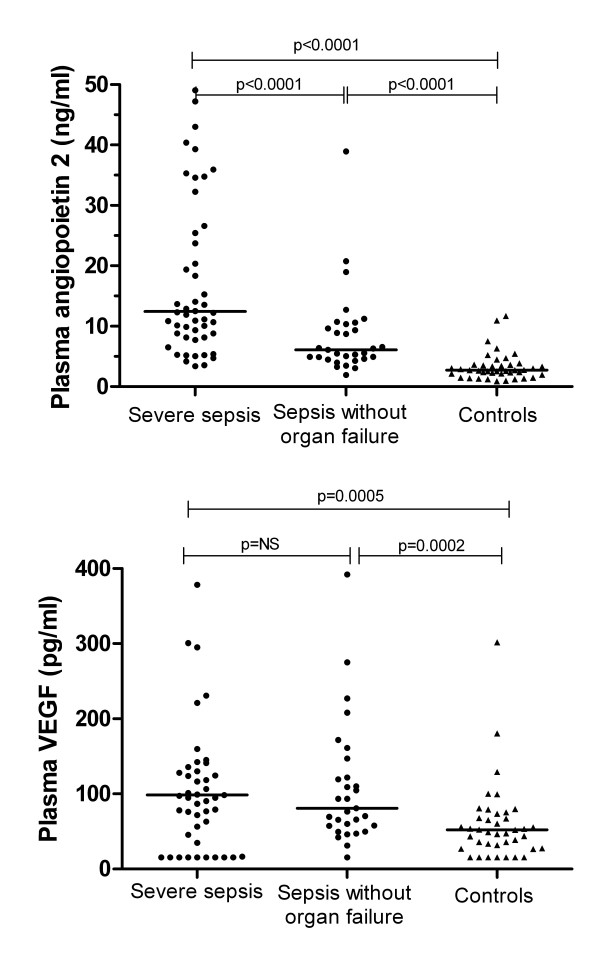
**Baseline plasma angiopoietin-2 and vascular endothelial growth factor across disease categories**. **(a) **Baseline plasma angiopoietin-2. **(b) **Vascular endothelial growth factor. Solid circles represent individual sepsis subjects and solid triangles represent control subjects. Horizontal lines through the data points represent group median values. *P *values indicate pairwise comparisons between groups as indicated. NS, not significant; VEGF, vascular endothelial growth factor.

Plasma Ang-2 concentrations were raised in sepsis in proportion to disease severity (Table 2 and Figure 1a). Median Ang-2 concentrations (ng/ml (interquartile range (IQR)) were two-fold higher in patients with severe sepsis (12.4 (8.5 to 33.4)), than in those with sepsis without organ failure (6.1 (5.0 to 10.4); *P *< 0.0001), and 4.5-fold higher than in controls (2.7 (2.2 to 3.6), *P *< 0.0001). The difference in Ang-2 between sepsis without organ failure and controls was also significant (*P *< 0.0001). VEGF was also raised in sepsis patients compared with controls (*P *= 0.0001, Table 2 and Figure 1b), but the difference in VEGF between severe sepsis and sepsis without organ failure was not significant.

**Table 2:** Baseline measurements according to disease category

	Severe sepsis	Sepsis without organ failure	Control	*P *value across all groups
**N**	52	31	41	
**Angiopoietin 2 (ng/ml)^a^**	12.44 (8.47-33.44)	6.11 (4.59-10.37)	2.71 (2.15-3.61)	0.0001^d^
**VEGF (pg/ml)^a^**	98.4 (56.4-142.6)	80.8 (57.5-147.3)	52.3 (31.8-73.5)	0.0007^d^
**Plasma ICAM-1 (ng/ml)^a^**	846 (523-1483)	501 (368-672)	323 (265-393)	0.0001^d^
**Plasma E-selectin (ng/ml)^a^**	200.5 (113-478)	87.0 (50.8-164.4)	38.4 (26.9-58.2)	0.0001^d^
**RH-PAT index^b^**	1.57 (1.44-1.71)	1.85 (1.67-2.03)	2.07 (1.93-2.22)	<0.0001^e^
**Plasma interleukin 6 (pg/ml)^a^**	385.1 (124.2-996.0)	148.3 (45.9-315.0)	5.0 (2.2-8.1)	0.0001^d^
**Plasma TNFα ≥ 2.8 pg/ml (n,%)^c^**	8 (22)	4 (14)	5 (17)	NS^f^

a. Median (Interquartile range).

b. Mean (95% confidence interval).

c. 2.8 pg/ml is the lower limit of detection for the assay used for TNFα.

d. By Kruskal-Wallis test.

e. By oneway analysis of variance.

f. By Fisher's exact test across all three groups.

ICAM-1, intra-cellular adhesion molecule-1; NS, not significant; RH-PAT, reactive hyperemia peripheral arterial tonometry; TNFα, tumor necrosis factor α; VEGF, vascular endothelial growth factor.

### Ang-2 and disease severity

Ang-2 correlated with sepsis severity (Table 3), as measured by Acute Physiology and Chronic Health Evaluation (APACHE) II score (r = 0.46, *P *< 0.0001), Sequential Organ Failure Assessment (SOFA) score (r = 0.58, *P *< 0.0001), number of organ failures (r = 0.48, *P *< 0.0001) and arterial lactate (r = 0.41, *P *= 0.003), whereas VEGF did not correlate with any of these parameters. As neutrophils release H_2_O_2_, we also examined the relation between neutrophil counts and plasma Ang-2 concentrations, and found no significant correlation (r = 0.16, *P *= 0.15).

**Table 3:** Correlations of baseline plasma angiopoietin-2 in sepsis patients

	Spearman's rho	P	n
***Endothelial function and activation***			
**RH-PAT index**	-0.38	<0.0001	74
**VEGF**	-0.04	NS	80
**ICAM-1**	0.58	<0.0001	83
**E-Selectin**	0.53	<0.0001	83
***Markers of inflammation***			
**IL-6**	0.57	<0.0001	66
**C-reactive protein**	0.16	NS	82
***Markers of disease severity***			
**SOFA score**	0.58	<0.0001	82
**APACHE II score**	0.46	<0.0001	83
**Number of organ failures**	0.48	<0.0001	83
**Arterial lactate**	0.41	0.003	51

APACHE II score, Acute Physiology and Chronic Health Evaluation II Score; ICAM-1, intra-cellular adhesion molecule-1; IL-6, interleukin 6; NS, not significant; RH-PAT, reactive hyperemia peripheral arterial tonometry; SOFA, Sequential Organ Failure Assessment score; VEGF, vascular endothelial growth factor.

### Ang-2 and NO-dependent microvascular reactivity

On univariate analysis, Ang-2 was inversely correlated with RH-PAT index, an estimate of endothelial NO bioavailability (r = -0.38, *P *< 0.0001), and positively correlated with markers of endothelial activation (ICAM-1 r = 0.58, *P *≤ 0.0001, E-selectin r = 0.53, *P *< 0.0001). VEGF did not correlate with endothelial NO bioavailability, endothelial activation or plasma Ang-2. The relationship between log Ang-2 and RH-PAT index remained significant after controlling for disease severity using SOFA score.

In a longitudinal analysis, plasma Ang-2 concentrations decreased significantly between day 0 (median (IQR) 10.16 (5.32 to 19.39)) and day two to four (8.72 (5.38 to 15.73), *P *= 0.01; Table 4). RH-PAT index increased over the same time period, but the change was not statistically significant (Day 0 mean index 1.67 (95% confidence interval (CI): 1.55 to 1.78), day two to four = 1.85 (1.70 to2.00)). In a mixed-effects linear regression model, increase in RH-PAT index over the first two to four days correlated significantly with fall in Ang-2 (r = 0.45, *P *< 0.0001); change in RH-PAT index over time did not correlate with VEGF, ICAM-1, E-selectin, SOFA score, or IL-6.

**Table 4:** Change in angiopoietin-2 and other variables over time

	Day 0	Day 2-4	*P *value^a^
**RH-PAT^b^**	1.67 (1.55-1.78)	1.83 (1.67-1.99)	NS
**SOFA score^c^**	4 (2-8)	3 (1-7)	0.07
**Ang-2 (ng/ml)^c^**	10.16 (5.32-19.39)	8.72 (5.38-15.73)	0.01
**VEGF (pg/ml)^c^**	94.8 (57.0-143.8)	73.6 (48.6-167.3)	NS
**ICAM-1 (ng/ml)^c^**	655.6 (448.0-1084.1)	694.0 (450.3-1258.1)	NS
**E-selectin (ng/ml)^c^**	154.5 (69.1-396.3)	120.3 (63.9-199.2)	<0.0001
**IL-6 (pg/ml)^c^**	224.4 (75.0-595.5)	58.1 (17.2-255.8)	<0.0001

a. Mean (95% confidence interval).

b. Median (Interquartile range).

c. Wilcoxon's paired signrank test.

Ang-2, angiopoietin-2; ICAM-1, intra-cellular adhesion molecule-1; IL-6, interleukin 6; NS, not significant; RH-PAT, reactive hyperemia peripheral arterial tonometry; SOFA, Sequential Organ Failure Assessment score; VEGF, vascular endothelial growth factor.

### Ang-2 and markers of inflammation

Plasma TNFα was below the lower limit of detection in the majority of patients in both the sepsis and control groups (Table 2). In those in whom it was detectable (≥ 2.8 pg/ml), there was no relation between Ang-2 and TNFα (r = 0.24, *P *= 0.44). Ang-2 correlated with IL-6 (r = 0.57, *P *< 0.0001), but not with C-reactive protein or white blood cell count.

### Multivariate analysis of correlates of angiopoietin-2

The variables included in the initial multivariate linear regression model, with log Ang-2 as the dependent variable, were: RH-PAT index, serum albumin, APACHE and SOFA scores, plasma concentrations of E-selectin, ICAM-1, IL-6 and IL-10, and peripheral blood platelet and white blood cell counts. The independent variables that remained significant in the final model, along with their β coefficients (95% CI) were: RH-PAT index (β = -0.35 (-0.64 to -0.06)), ICAM-1 (ng/ml, β = 3.8 × 10^-4 ^(1.4 to 6.3 × 10^-4^)), IL-6 (pg/ml, β = 1.7 × 10^-4 ^(0.05 to 2.9 × 10^-4^)), platelet count (× 10^9^/L, β = -2 × 10^-3 ^(-3.2 to -0.90 × 10^-3^)), and white blood cell count (× 10^9^/L, β = 3.2 × 10^-2 ^(1.1 to 5.2 × 10^-2^)).

### Outcomes

The median (IQR) length of stay in the ICU among all sepsis patients was 5.4 (3.0 to 8.4) days, and this was significantly correlated with baseline Ang-2 (r = 0.30, *P *= 0.03). Of the 83 patients with sepsis, only 8 had died at 28-day follow up (10%). Seven of these were from the severe sepsis group (28-day mortality 13%) and one was from the sepsis without organ failure group (mortality 3%). Baseline levels of Ang-2 were not significantly different (*P *= 0.32) in those with fatal (11.46 (7.09 to 45.12)) and non-fatal outcomes (10.04 (5.26 to 18.96)).

## Discussion

Plasma Ang-2 concentrations are raised in patients with sepsis, in proportion to disease severity and endothelial cell activation, and are inversely associated with estimated endothelial NO bioavailability both at baseline and during recovery. This finding supports the hypothesis that impaired endothelial NO bioavailability in sepsis leads to increased exocytosis of WPBs, release of Ang-2, and thus to further endothelial cell sensitization and activation. This hypothesis is also supported by recent findings in patients with severe malaria, where an increase in endothelial NO bioavailability over time (also measured by RH-PAT) was significantly associated with falling plasma Ang-2 levels [23].

Although we demonstrate for the first time the relation between estimated endothelial NO bioavailability and plasma Ang-2 concentrations in sepsis, there is substantial recent evidence underpinning this hypothesis. *In vitro*, NO is the only substance demonstrated to reduce exocytosis of WPBs and release of Ang-2 apart from high concentrations of H_2_O_2 _[13,14]. NO reduces WPB exocytosis by facilitating the S-nitrosylation of N-ethyl-malemide sensitive factor (NSF), which results in the inability of the WPB membrane to fuse with the plasma membrane [2,12,13]. Furthermore, contrary to previously accepted theories, there is increasing evidence that systemic NO production is normal or decreased rather than increased in sepsis [18,19], and that sepsis is a state of imbalance between the endothelial and inducible isoforms of NO synthase in the microvasculature, resulting in a relative deficiency of endothelial NO [24,25]. The fact that non-specific NO inhibitors increase mortality in patients with sepsis [26] supports this idea, and it is possible that increased Ang-2 release is one of the mechanisms underlying this finding.

Clinical studies investigating the endothelium in sepsis commonly use circulating markers of endothelial cell activation as a surrogate measure of endothelial function. We have previously shown that ICAM-1 and E-selectin, two of the most commonly used markers of endothelial activation in sepsis, do not correlate with endothelial function as measured by RH-PAT [15]. In contrast, Ang-2 correlates with endothelial function as measured by RH-PAT, both at baseline and longitudinally. Thus Ang-2 is a more meaningful biomarker of endothelial cell function in sepsis than currently used surrogate measures.

Endothelial cell activation in sepsis increases vascular leak, triggers a pro-coagulant state, up-regulates adhesion molecule expression, and further drives the inflammatory response [27]. Together, these processes cause regional hypoperfusion and acute organ dysfunction. By mediating autocrine activation of local endothelial cells, Ang-2 may exacerbate tissue hypoperfusion and inflammation, providing a plausible mechanism for its independent association with organ failure and mortality in sepsis [7].

*In-vitro *studies of Ang-2 demonstrate that in the absence of Ang-2, a TNFα concentration of 40 pg/ml or more is required to independently activate endothelial cells, whereas in the presence of Ang-2 at a concentration of 2000 pg/ml, a TNFα concentration of 5 pg/ml or more is able to cause endothelial activation [28]. Plasma TNFα levels in sepsis patients in this study were relatively low, with only 1 of 69 sepsis patients having TNFα levels of 40 pg/ml or more, similar to the low levels reported in other sepsis studies [29,30]. The concentrations of Ang-2 in this and other human sepsis studies [4,5,7] are higher than those used in *in-vitro *studies, and may sensitize endothelial cells to lower concentrations of TNFα. Furthermore, local microvascular TNFα concentrations may be higher than we and others have found in plasma. Nevertheless, taken together our results support the hypothesis that in sepsis, Ang-2 sensitizes endothelial cells to the effects of cytokines that may otherwise cause only minimal or no endothelial activation [28].

The factors triggering Ang-2 release from WPBs in sepsis are not known. Thrombin [12], VEGF [31] and, in some [31,32], but not other studies [4,12], TNFα, cause WPB release *in-vitro*. However, we found that neither TNFα nor VEGF correlated with Ang-2. Although we found a strong independent association between IL-6 and Ang-2, IL-6 has not been shown to cause exocytosis of WPBs or secretion of Ang-2 *in vitro*. IL-6 is an important pro-inflammatory cytokine and correlates with disease severity in sepsis. Bacterial lipopolysaccharide increases Ang-2 levels [33] and drives IL-6 expression [30], and such factors may account for this association.

Although Ang-2 correlated with length of stay in this study, it did not correlate with mortality. Despite a median APACHE II score of 19, and a consequent predicted mortality of 34.8% [34], there were few deaths in our study (8 in total, 13% within the severe sepsis group). This is consistent with the previously reported low mortality from severe sepsis in our ICU [35], and suggests that our study was under-powered to examine the relation between Ang-2 and mortality. However, in studies with higher numbers of deaths, Siner and colleagues and Kumpers and colleagues both found a clear association between plasma Ang-2 levels and risk of mortality [7,8].

Although we did not directly measure endothelial cell NO concentrations (which is not possible in septic patients), RH-PAT index is an indirect measurement of NO bioavailability and is at least 50% NO-dependent in healthy volunteers [16]. Other methods of measuring NO in patients with sepsis, such as plasma NO metabolites, are not specific to the endothelium and are confounded by nitrate retention in renal failure [36]. Thus it is not possible to directly confirm the relation between endothelial NO bioavailability and plasma Ang-2 using currently available methods in humans with sepsis.

The correlation between Ang-2 and RH-PAT index was statistically significant but was not strong. We cannot exclude an alternative explanation for the inverse association between Ang-2 and endothelial NO bioavailability: that increased Ang-2 release in sepsis leads to decreased NO bioavailability as a consequence of upregulated endothelial cell inflammation and superoxide-mediated NO quenching. Nevertheless the clear *in vitro *evidence for NO as the major inhibitor of WPB exocytosis and Ang-2 release, and the findings in other disease settings such as malaria [23], make it more likely that impaired NO bioavailability is a significant contributor to Ang-2 release in sepsis.

vWF is co-packaged with Ang-2 in WPBs but is also released by activated platelets, and is thus less specific for endothelial cells. Although not measured in our study, plasma vWF activity is known to be increased in patients with sepsis, and to correlate with mortality [37]. Like other markers of endothelial cell activation, vWF has not been compared with measures of endothelial NO bioavailability in sepsis. However, in non-septic patients with risk factors for cardiovascular disease, vWF is raised, correlates with endothelial activation as measured by E-selectin [38], and is inversely proportional to endothelial NO bioavailability as estimated by flow-mediated dilatation of the brachial artery [39]. Furthermore, plasma vWF is raised in proportion to plasma Ang-2 in patients with sepsis and acute lung injury [11]. Because our results suggest that impaired endothelial NO bioavailability exacerbates WPB release, they provide a plausible explanation for the increase in both Ang-2 and vWF in patients with sepsis.

## Conclusions

In conclusion, Ang-2 is raised in sepsis in proportion to disease severity and correlates with endothelial activation and inversely with NO-dependent microvascular reactivity, both at baseline and over the first two to four days of treatment. This suggests that decreased endothelial NO bioavailability may contribute to Ang-2 release by reducing negative feedback on WPBs, thus augmenting endothelial cell activation and contributing to organ dysfunction. Adjunctive therapies which improve endothelial NO, decrease WPB release, or antagonise Ang-2 may have roles in reducing organ dysfunction and improving mortality in sepsis.

## Key messages

• Plasma concentrations of Ang-2, an angiogenic peptide, have been shown to be raised in patients with sepsis and to correlate with organ failure and mortality, but the underlying mechanisms are unclear.

• *In-vitro*, NO inhibits Ang-2 release from endothelial cells. In this study, plasma concentrations of Ang-2 in septic patients were raised in proportion to disease severity, and were inversely proportional to estimated endothelial NO bioavailability.

• Decreased endothelial NO bioavailability in sepsis may be the mechanism for raised Ang-2 concentrations, thus contributing to capillary leak and organ dysfunction.

• Adjunctive therapies that improve endothelial NO or antagonise Ang-2 may have roles in reducing organ dysfunction and improving mortality in sepsis.

## Abbreviations

Ang-2: angiopoietin-2; APACHE: Acute Physiology and Chronic Health Evaluation; CBA: cytokine bead array; CI: confidence interval; ELISA: enzyme-linked immunosorbent assay; H_2_O_2_: hydrogen peroxide; ICAM-1: intra-cellular adhesion molecule-1; IL: interleukin; IQR: interquartile range; NSF: N-ethyl-malemide sensitive factor; NO: nitric oxide; RH-PAT: reactive hyperemia peripheral arterial tonometry; SIRS: systemic inflammatory response syndrome; SOFA: Sequential Organ Failure Assessment; TNFα: tumour necrosis factor α; VEGF: vascular endothelial growth factor; vWF: von Willebrand factor; WPB: Weibel Palade bodies.

## Competing interests

DC has received research support (as equipment) from Itamar Medical, the manufacturer of the RH-PAT device, and has received speaker's fees (less than US$1000 per year) for speaking at Itamar-sponsored educational events. The other authors have no competing interests.

## Authors' contributions

Study Design was performed by JSD, NMA, TWY, DPS and DSC. JSD and DPS contributed to patient recruitment. The data was processed by JSD, KP and TW, and was analysed by JSD. Laboratory sample processing was performed by KP and TW. The manuscript was drafted by JSD and NMA. All authors had access to all data and contributed to the final draft of the paper. All authors read and approved the final manuscript.
